# *In situ* synthesis of SO_3_H supported Fe_3_O_4_@resorcinol–formaldehyde resin core/shell and its catalytic evaluation towards the synthesis of hexahydroquinoline derivatives in green conditions

**DOI:** 10.1039/d0ra06972h

**Published:** 2020-11-13

**Authors:** Aliyeh Barzkar, Alireza Salimi Beni

**Affiliations:** Department of Chemistry, Faculty of Science, Yasouj University Yasouj 75918-74831 Iran salimibeni@yu.ac.ir alirezasalimi7173291@gmail.com

## Abstract

A novel spherically shaped core@double-shell acidic nanocatalyst (Fe_3_O_4_@SiO_2_@RF–SO_3_H) [RF: resorcinol–formaldehyde resin] was prepared *in situ* and completely characterized using X-ray diffraction, Fourier transform infrared spectroscopy, vibrating sample magnetometry, energy dispersive X-ray spectroscopy, thermogravimetric analysis, transmission electron microscopy and field-emission scanning electron microscopy. The concentration of H^+^ loaded on the Fe_3_O_4_@SiO_2_@RF was reported to be 1.3 mmol g^−1^. The well-defined Fe_3_O_4_@SiO_2_@RF–SO_3_H core–shell heterostructures exhibited high stability, efficient recyclability (10 cycles), and promoted catalytic activity for one-pot condensation reaction between the aromatic aldehydes, dimedone, malononitrile, and ammonium acetate for the synthesis of hexahydroquinoline derivatives.

## Abstract 2

A novel core@double-shell acidic nanocatalyst (Fe_3_O_4_@SiO_2_@RF–SO_3_H) was prepared, characterized and applied in catalytic one-pot condensation between aromatic aldehydes, dimedone, malononitrile, and ammonium acetate for synthesis of hexahydroquinoline derivatives.

## Graphical abstract

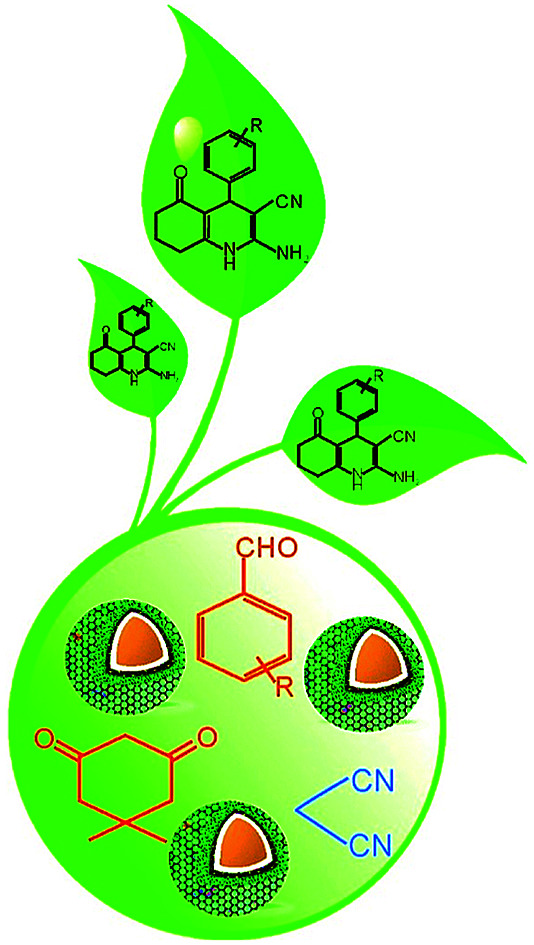

## 1. Introduction

Recently, quinolines and their derivatives have attracted considerable attention in organic chemistry due to their pharmacological significance. These compounds can be found in many natural products because of their biological properties. They are also benefited from favorable antibacterial, anti-fungal, antioxidant, anti-cancer and anticonvulsant properties.^1,2^ Quinoline and its derivatives are generally synthesized from four compounds using a one-step reaction of Hantzsch *via* a long cyclocondensation of aldehydes, β-keto ester, ammonia, and relevant catalyst. To improve the production efficiency of quinoline and its derivatives, several catalysts, such as Yb trifluoromethanesulfonate (OTf)_3_,^3^ K_7_[PW_11_CoO_40_],^4^*p*-toluenesulfonic acid (*p*-TSA),^5^ HClO_4_–SiO_2_,^6^ (Sc(OTf)_3_),^7^ cerium(iv) ammonium nitrate,^8^ and iron(iii) fluoride (FeF_3_),^9^ have been developed. All these catalysts suffer from several drawbacks, including difficult separation from the reaction medium, high cost, and the use of toxic organic solvents. An environmentally friendly and one-step synthesis approach, which involves the use of four compounds (*i.e.* aryl aldehydes, dimedone, ammonium acetate, and malononitrile), has been recently developed in the presence of a heterogeneous catalyst.^10–12^

The advancements in magnetic nanoparticles (MNPs)-based heterogeneous catalysts have opened new horizons for the development of modern science because of their unique features such as cost-effectiveness, non-toxicity and convenient separation with an external magnetic field. MNPs can be recovered and reused many times, which makes them more economical.^13,14^ MNPs, however, have several weaknesses among which low stability in the acidic and alkaline conditions, easy oxidization under humid atmospheric conditions, and agglomeration can be mentioned.^15,16^ These limitations could be overcome through surface modification with stable materials such as silica, carbon and polymer.^17–20^ The MNPs surface modification approaches can be divided into two categories: coating with inorganic materials (*e.g.* silica and metal oxides) and organic coatings containing carbon and polymers (*e.g.* resorcinol–formaldehyde resin).^18,21^ Resorcinol–formaldehyde resin has excellent characteristics, thus making it an appropriate candidate for stabilization. It can serve as a good precursor for carbon source; moreover, it has high chemical stability and hydrophilicity. To date, the use of resorcinol–formaldehyde resin in the synthesis of core–shell and yolk–shell structures has gained considerable interest.^22,23^ In some cases, the Stöber method was used for the sequential hydrolysis and concentration of organo-silanes^24^ to prepare the silica colloidal spheres as a reactive linking layer on the magnetic substrates.^25,26^

The magnetite based core/shell nanostructures have been studied for more than two decades due to their use in various fields such as catalysis,^27,28^ drug carriers,^29,30^ lithium batteries,^31,32^ sensors,^33,34^ and energy storage^35,36^, adsorbents.^37–39^ The core–shell structures containing magnetite as the core and the resorcinol–formaldehyde (RF), glucose, and dopamine as the shell can be used for substrate stabilization. Resorcinol–formaldehyde have attractive properties such as good stability, high surface area, low cost, remarkable electrical conductivity, controllable structure, and outstanding thermal and mechanical properties.^40–42^ Several core–shell catalysts, including Fe_3_O_4_@polydopamine,^43^ AgBr@SiO_2_@RF,^44^ noble metal@RF,^21^ Fe_3_O_4_@RF/Cu_2_O,^45^ Fe_3_O_4_@CFR-PdNPs, and Fe_3_O_4_@FR@graphene-oxide-PdNPs,^46^ have been developed in this category. An effective approach to increase the catalytic activity of these nanocomposites is to stabilize their functional groups by grafting and sol–gel methods. Sulfuric acid grafting on the surface of the core shells has several unique advantages, including large surface area, large pore volume, and easy functionalization. In particular, the core/shell structures with two shells is the best strategy to protect the magnetic core against oxidation and acidic conditions. Furthermore, the presence of acidic groups on the surface can improve its catalytic performance; moreover, sulfuric acid is a good Brønsted acid for the catalysis of several reactions (such as aldol condensations, acylations, nucleophilic additions, and hydrolysis).^20,47,48^

By increasing active sites in catalysts, especially in heterogeneous catalysts, the number of reactive sites and catalytic efficiency will be increased, thus providing additional active sites in the structure of the catalyst and can save the catalyst amount and the time-consumption. Regarding the importance of magnetic nanoparticles, this study is the first report on nanocatalysts modified by silica and resorcinol–aldehyde with high surface area and double-shell core–shell structure (Fe_3_O_4_@SiO_2_@RF–SO_3_H). In this manner, the MNPs surface was protected against oxidation, thus making them suitable for use as a stabilizing agent for sulfonic acid. In this case, the modification of the surface of nanoparticles with organic shells, in addition to protecting the magnetic properties of these Fe_3_O_4_ NPs, increases the surface hydrophobicity due to the presence of organic groups. This hydrophobicity increases the application of core–shell-structured organic coated NPs in catalytic processes. This nanostructure was characterized by various analysis methods; it was then employed as a new and useful acidic nanocatalyst for synthesizing hexahydroquinoline derivatives under solvent-free conditions at 40 °C.

## 2. Experimental section

### 2.1. Materials and methods

Chemicals such as tetraethyl orthosilicate (TEOS), resorcinol, formaldehyde, ammonia solution (25–28%), FeCl_3_·6H_2_O, FeCl_2_·4H_2_O, ethanol, HCl, malononitrile, dimedone, and all applied aldehydes were purchased from Merck, Fluka, and Aldrich. All solvents were dried and purified before application in the reactions. Purification of reaction products was performed *via* TLC on silica gel polygram SILG/UV 254 plates. The melting points were determined by a Barnstead Electrothermal (BI 9300) apparatus. FTIR spectra were obtained using an FT-IR JASC0-680 spectrometer. NMR spectra were obtained with a Bruker 400 MHz Ultrashield spectrometer at 400 MHz (^1^H) and 100 MHz (^13^C) using CDC1_3_ or DMSO-d_6_ as the solvent with TMS as the internal standard. Filed-emission scanning electron microscopy (FESEM) analysis was conducted by a Philips, XL30 emission electron microscope. Thermogravimetric analysis (TGA) was performed by NETZSCH STA 409 PC/PG from room temperature to 800 °C.

### 2.2. Preparation of Fe_3_O_4_

Magnetic nanoparticles were prepared as follows: FeCl_2_·4H_2_O 2 g and FeCl_3_·6H_2_O 5.2 g was mixed in 25 mL of HCl (1 N). Then, 250 mL of NaOH solution was dropwise added for 20 min under N_2_ purging at 80 °C. Finally, the black precipitates were separated by an external magnet, washed several times with distilled water and dried at 40 °C.^49^

### 2.3. Preparation of Fe_3_O_4_ core@SiO_2_ shell

To coat a SiO_2_ layer around the Fe_3_O_4_ nanoparticle, the Stöber method was used. In a typical procedure, 0.15 g of as-prepared Fe_3_O_4_ NPs was monodispersed in 60 mL of distilled water/ethanol (1 : 2) for 30 min. Then, 10 mL of NH_4_OH and 2 mL of TEOS was added to the mixture in a round-bottom flask and stirred at room temperature for 6 h. Finally, the crude product was separated by an external magnet and washed several times with water and ethanol and dried at 60 °C for 12 h.^50^

### 2.4. Preparation of Fe_3_O_4_@SiO_2_@RF

Polymeric resorcinol-formaldehyde shell was coated on the Fe_3_O_4_@SiO_2_ surface through the following steps: 0.3 g of Fe_3_O_4_@SiO_2_ nanostructure, 0.9 g of formaldehyde, and 28 mL of distilled water were transferred in a round-bottom flask and fully dispersed under ultrasound waves for 90 min. Then, 1.4 g of resorcinol, 120 mL of ethanol and 0.4 mL of ammonium hydroxide were added to the mixture. To complete the reaction, after 30 min of stirring at 35 °C, 2 mL of formaldehyde solution was added and the mixture was stirred for additional 6 h at room temperature. The reaction mixture was aged at room temperature overnight without complete polymerization. Finally, the product was collected by an external magnet and washed several times by water and ethanol and dried in an oven at 60 °C for 12 h.^51^

### 2.5. Preparation of Fe_3_O_4_@SiO_2_@RF–SO_3_H

**Fig. 1:**
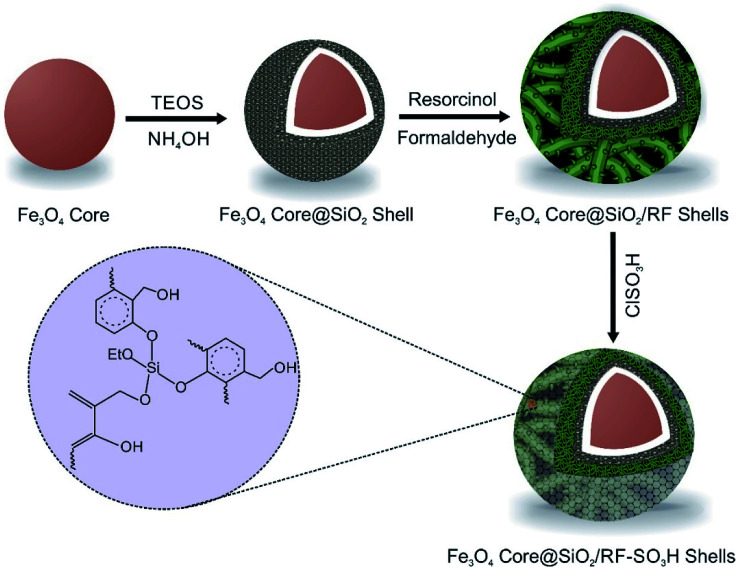
Illustration for the synthetic steps of Fe_3_O_4_@SiO_2_@RF–SO_3_H.

The Fe_3_O_4_@SiO_2_@RF–SO_3_H catalyst preparation involved the replacement of the hydroxyl groups of Fe_3_O_4_@SiO_2_@RF with sulfuric acid groups. For this purpose, 1.0 g of Fe_3_O_4_@SiO_2_@RF core-double shell nanostructure was dispersed by ultrasonic waves into 40 mL of chloroform in a two-neck round-bottom flask. Then, 1 mL of chlorosulfonic acid was dropwise added to the reaction mixture and stirred for 3 h at room temperature under an argon atmosphere. Finally, the magnetic product was separated with an external magnet and washed several times with chloroform and ethanol and dried at 40 °C. All stages for the production of Fe_3_O_4_@SiO_2_@RF–SO_3_H are presented in Fig. 1.

### 2.6. Determination of acidity of the Fe_3_O_4_@SiO_2_@RF–SO_3_H nanocatalyst

The amount of sulfuric acid loaded on the nanostructure surface was calculated by back titration by 0.5 N HCl. To this end, 0.3 g of Fe_3_O_4_@SiO_2_@RF–SO_3_H nanocatalyst was added into 15 mL of NaOH solution (0.2 N) and stirred at room temperature for 24 h; then, the extracted NaOH was titrated by HCl. According to this experiment, the loading of H^+^ was 1.3 mmol g^−1^.

### 2.7. General procedure for the preparation of hexahydroquinoline

Aldehydes (1 mmol), dimedons (1 mmol), ammonium acetate (1 mmol), malononitrile (1 mmol), and Fe_3_O_4_@SiO_2_@RF–SO_3_H nanocatalyst (0.9 mol%) were thoroughly mixed in a round-bottom flask and heated to 40 °C under continuous stirring. Reaction completion was confirmed by TLC and then the products were dissolved in hot ethanol (8 mL) and Fe_3_O_4_@SiO_2_@RF–SO_3_H was separated by an external magnet. The solvent was then evaporated and crude products were recrystallized and purified in ethanol, followed by IR, ^1^H NMR, and ^13^C NMR characterization.

## 3. Results and discussions

### 3.1. Materials characterization

**Fig. 2:**
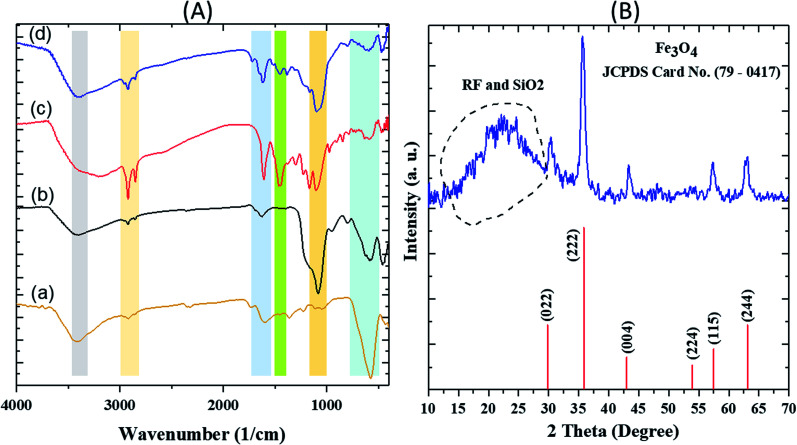
FT-IR spectra of (A) (a) Fe_3_O_4_ (b) Fe_3_O_4_@SiO_2_ (c) Fe_3_O_4_@SiO_2_@RF and (d) Fe_3_O_4_@SiO_2_@RF–SO_3_H and (B) XRD pattern of Fe_3_O_4_@SiO_2_@RF–SO_3_H sample.

The FT-IR spectra of various steps involved during the synthesis of the nanocatalyst are first described. In the FTIR spectrum of Fe_3_O_4_, a strong and evident peak at 585 cm^−1^ can be related to the Fe–O vibration. The vibrations of the hydroxyl group at 1630 cm^−1^ (bending vibration) and 3405 cm^−1^ (anti-symmetric stretching vibration) confirmed the successful preparation of Fe_3_O_4_. For Fe_3_O_4_@SiO_2_, a wide absorption band at 1083 cm^−1^ is related to the Si–O–Si symmetric stretching vibrations, Si–O bands could be observed in the range of 460–789 cm^−1^. The vibrations of hydroxyl group related to the SiO_2_ at 3434 cm^−1^ are easily observable. In the spectrum of Fe_3_O_4_@SiO_2_@RF, intensity of the Si–O–Si and Si–O peaks decreased due to the presence of RF polymer. The absorption bands at 1420 cm^−1^ and 1635 cm^−1^ correspond to the aromatic rings in the RF. The successful stabilization of sulfuric acid groups on the surface of the Fe_3_O_4_@SiO_2_@RF can be confirmed based on the vibrations at 1000–1300 cm^−1^ (Fig. 2A).

The XRD pattern of Fe_3_O_4_@SiO_2_@RF–SO_3_H is depicted in Fig. 2B. The diffraction peaks at 2*θ* = 30.0°, 35.5°, 43.2°, 53.8°, 57.2°, and 62.6° can be attributed to 022, 222, 004, 224, 114 and 244 crystal planes, respectively, confirming the crystalline structure of the magnetic Fe_3_O_4_.^52^ The broadband peak observed in the range of 15°–30° could be assigned to SiO_2_ and RF amorphous layers.^53,54^

**Fig. 3:**
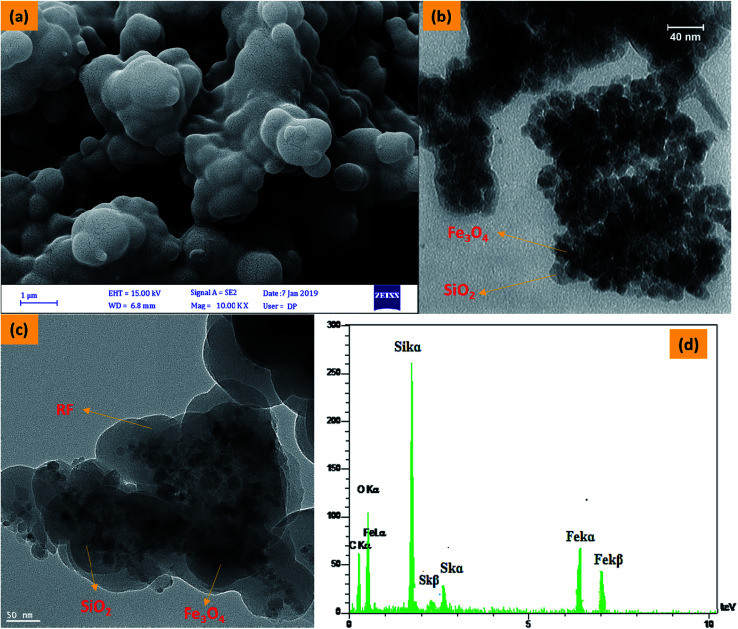
FESEM image of Fe_3_O_4_@SiO_2_@RF–SO_3_H (a), TEM image of Fe_3_O_4_@SiO_2_ (b) and Fe_3_O_4_@SiO_2_@RF–SO_3_H (c) and EDS spectra of the Fe_3_O_4_@SiO_2_@RF–SO_3_H (d).

FESEM (Fig. 3a) images of Fe_3_O_4_@SiO_2_@RF-SO_3_H show the nano spherical shape of the samples and confirm the formation of a quite rough structure. TEM (Fig. 3b) and HRTEM (Fig. 3c) was used to visualize the formation of Fe_3_O_4_@SiO_2_ core–shell and Fe_3_O_4_@SiO_2_@RF–SO_3_H core-double-shell nanostructure, respectively. Fig. 3b shows the nanoparticle-assembled cluster core of ∼30 nm and uniform carbon shell of ∼20 nm around the Fe_3_O_4_ NPs. Subjected to the successive precipitation polymerization through the binding of RF, the magnetic composite nanoparticles were densely encapsulated by the RF of ∼70 nm (Fig. 3c). A close inspection of the outer shell in Fig. 3c indicates a rough surface with discontinuous coverage on the Fe_3_O_4_@SiO_2_ shell. Energy-dispersive X-ray (EDAX) spectra (Fig. 3d) confirms the presence of Fe, Si, O, C and S, thus proving the existence of silica and polymeric layers on the surface of magnetite nanoparticles and the stabilization of acidic groups of sulfuric acid on the core–shell surface.

**Fig. 4:**
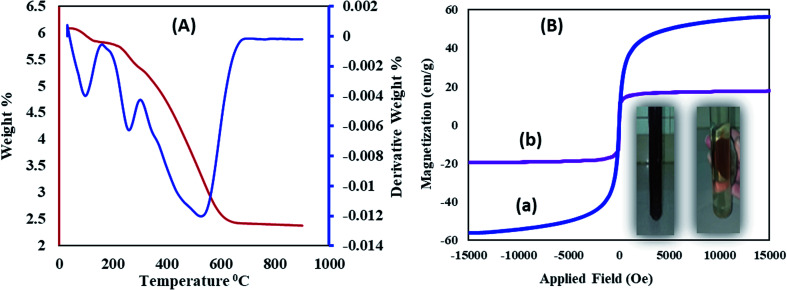
(A) Thermal gravimetric analysis of Fe_3_O_4_@SiO_2_@RF–SO_3_H and (B) VSM analysis of (a) Fe_3_O_4_ and (b) Fe_3_O_4_@SiO_2_@RF–SO_3_H.

Thermogravimetric and differential thermal analyses of the Fe_3_O_4_@SiO_2_@RF–SO_3_H (Fig. 4A) confirm the thermal stability of the as-prepared sample. The presence of fixed groups can be interpreted as follows: the initial weight loss below 200 °C is related to the removal of adsorbed solvents and hydroxyl groups; however, the second weight loss is observed in the temperature range of 200 to 300 °C, which reflects the removal of sulfonic acid groups. The last weight loss can be assigned to the thermal decomposition of the nanostructure at 350–700 °C, thus confirming the stability of the synthesized nanostructure.

Vibrating sample magnetometer (VSM) curve of (Fig. 4B) revealed that the saturation magnetization of Fe_3_O_4_ nanoparticles was approximately 60 emu g^−1^, which decreased to 17.05 emu g^−1^ after SiO_2_@RF–SO_3_H coating. This decrease in the saturation magnetization could be due to the formation of silicone and polymer coatings on the surface of Fe_3_O_4_ nanoparticles, which confirms the successful stabilization of the acidic groups on the Fe_3_O_4_@SiO_2_@RF surface. An image for the collection of Fe_3_O_4_@SiO_2_@RF–SO_3_H catalyst by an external magnet is in the inset of Fig. 4B. Magnetic property is one of the best and most effective advantages of this novel nanocatalyst, which contributes in its easy separation and cost-effectiveness.

### 3.2. Optimization of hexahydroquinoline synthesis using Fe_3_O_4_@SiO_2_@RF–SO_3_H

The catalytic reaction for the hexahydroquinoline preparation was performed in the presence of Fe_3_O_4_@SiO_2_@RF–SO_3_H. Before the synthesis of these derivatives, the ideal conditions were optimized in the blank reaction. To identify the best reaction conditions, the reactions of benzaldehyde with malononitrile, dimedons, and ammonium acetate were considered as blank reactions. The effects of various parameters such as catalyst loading, solvent type, and temperature were then optimized (Table 1). The reaction took a long time (4 h) in the absence of Fe_3_O_4_@SiO_2_@RF–SO_3_H, which confirms the importance of the presence of Fe_3_O_4_@SiO_2_@RF–SO_3_H. A increase in the amount of catalyst from 0.18 to 0.90 mol% led to an enhance in the product yield; however, its additional increase from 0.9 to 1.8 mol% did not change the yield (Table 1, entries 1–5). Therefore, 0.9 mol% of the catalyst was selected as the optimum catalyst amount (Table 1, entry4). Several solvents were used to identify the optimal solvent type (Table 1, entries 9–13). It was observed that nonpolar solvents such as toluene and acetonitrile resulted in lower yields compared to polar and protic solvents (*e.g.* water and ethanol). In the solvent-free condition, 98% yield was observed (Table 1, entries 4). The reaction temperature was studied for selecting the appropriate temperature conditions (room temperature, 30, 40, and 60 °C). A comparison of obtained yields showed that the best yield can be achieved at 40 °C; therefore, this temperature was selected as the optimum temperature conditions (Table 1, entries 4–8). To show the neat effect of the supported sulfuric acid sites during this catalytic process, the catalytic activity of Fe_3_O_4_@SiO_2_ and Fe_3_O_4_@SiO_2_@RF nanomaterials in the model reaction was studied; the results were compared with those of the present catalyst. Interestingly, the results showed that in the presence of both sulfuric acid and free nanomaterials, no yield of the desired product was obtained under the same conditions and time as that of the Fe_3_O_4_@SiO_2_@RF–SO_3_H, thus confirming that the synthesis of hexahydroquinoline process is primarily catalyzed by the supported –SO_3_H moieties.

**Table 1:** Optimization conditions of Fe_3_O_4_@SiO_2_@RF–SO_3_H catalyst amount, solvent type and temperature on blank reaction^a^

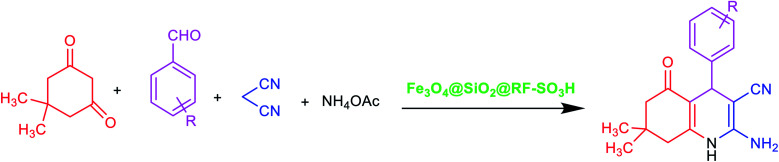					
Entry	Solvent	Catalyst loading (mol%)	*T* (°C)	*t* [min]	Yield^b^ [%]
1	—	—	40	240	—
2	—	0.18	40	35	>90%
3	—	0.54	40	25	>95%
**4**	**—**	**0.9**	**40**	**15**	**>98%**
5	—	1.8	40	10	>98%
6	—	0.9	RT	50	60%
7	—	0.9	30	45	90
8	—	0.9	60	10	>98%
9	H_2_O	0.9	40	25	90
10	EtOH	0.9	40	25	93%
12	Toluene	0.9	40	35	50%
13	CH_3_CN	0.9	40	40	58%
14	—	Fe_3_O_4_@SiO_2_^c^	40	60	—
15	—	Fe_3_O_4_@SiO_2_@RF^d^	40	60	—

a Reaction conditions: benzaldehyde (1 mmol), dimedone (1 mmol), malononitrile (1 mmol), ammonium acetate (1 mmol) and Fe_3_O_4_@SiO_2_@RF–SO_3_H nanocatalyst (0.9 mol%).

b Isolated yields.

c 0.02 g of Fe_3_O_4_@SiO_2_ was used as a catalyst.

d 0.02 g of Fe_3_O_4_@SiO_2_@RF was used as a catalyst.

### 3.3. Synthesis of hexahydroquinoline derivatives at the presence of Fe_3_O_4_@SiO_2_@RF–SO_3_H

After optimizing the blank reaction conditions, certain benzaldehyde derivatives with electron-donating groups and electron-accepting groups were studied. The results indicated a short reaction time and high efficiency for both types of groups. This indicates that the position and type of substitution did not have a significant impact on the reaction process, thus confirming the effective performance of the new Fe_3_O_4_@SiO_2_@RF–SO_3_H nanocatalyst for synthesizing hexahydroquinoline derivatives (Table 2). FTIR, ^1^H NMR, and ^13^C NMR spectral data were used to identify the structure of the product.

**Table 2:** Synthesis of hexahydroquinoline derivatives in the presence of Fe_3_O_4_@SiO_2_@RF–SO_3_H nanocatalyst at 40 °C under solvent-free condition^a^

Entry	R	Time	Yield^b^ (%)	MP	Ref.
1	H	15	95	285–286	286 (ref. 12)
2	4-CH_3_	18	92	290–292	294–295 (ref. 12)
3	4-OCH_3_	20	91	290–291	289–293 (ref. 12)
4	2-OCH_3_	21	91	288–289	—
5	2-OH	22	90	290–291	—
6	4-Isopropyl	20	93	289–290	—
7	4-NO_2_	12	98	280–292	290–293 (ref. 12)
8	3-NO_2_	13	97	282–284	282–283 (ref. 12)
9	4-CN	13	97	287–290	—
10	4-Cl	14	92	289–291	290–291 (ref. 10)
11	2,4-Cl	13	94	290–293	—
12	4-Br	13	92	296–298	295–296 (ref. 10)
13	3-Br	12	91	292–294	293–294 (ref. 12)

a Reaction conditions: benzaldehyde (1 mmol), dimedone (1 mmol), malononitrile (1 mmol), ammonium acetate (1 mmol) and Fe_3_O_4_@SiO_2_@RF–SO_3_H nanocatalyst (0.9 mol%).

b Isolated yields.

### 3.4. Reusability and recovery of Fe_3_O_4_@SiO_2_@RF–SO_3_H

**Fig. 5:**
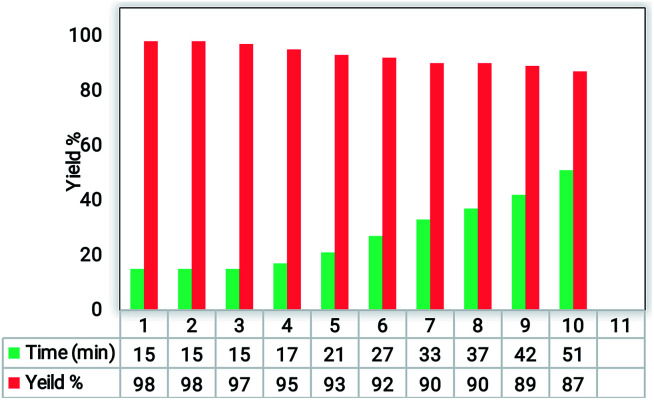
Recoverability and reusability results of the Fe_3_O_4_@SiO_2_@RF–SO_3_H nanocatalyst.

The recovery and reusability test is a simple and easy approach to assess the heterogeneity and performance of a catalyst. To study the recycling ability and reusability of the Fe_3_O_4_@SiO_2_@RF–SO_3_H nanocatalyst, the reaction between benzaldehyde, dimedone, malononitrile, and ammonium acetate was performed under optimum conditions. After completion of the reaction (detected by TLC), the Fe_3_O_4_@SiO_2_@RF–SO_3_H catalyst was collected by an external magnet and washed with hot ethanol. Fe_3_O_4_@SiO_2_@RF–SO_3_H was again used in the same reaction under the same conditions for 10 cycles and did not show any efficiency variations (see Fig. 5).

### 3.5. Comparisons with literature

The performance and efficiency of the synthesized acidic Fe_3_O_4_@SiO_2_@RF–SO_3_H nanocatalyst was compared with the other synthesized catalysts in Table 3. Accordingly, all previously reported catalysts were among the components of our blank reaction. All of them required higher temperatures, took longer time to react, and had lower yields and reaction cycles than Fe_3_O_4_@SiO_2_@RF–SO_3_H. The superior performance of Fe_3_O_4_@SiO_2_@RF–SO_3_H nanocatalyst, (*e.g.* its shorter reaction time, solvent-free conditions, high efficiency, and higher response cycle) can be assigned to its ease of recyclability, structural features (*e.g.* high surface area, surface uniformity), and high stability of functionalized acid groups.

**Table 3:** Comparison of catalytic activity of the Fe_3_O_4_@SiO_2_@RF–SO_3_H nanocatalyst with several known catalysts in the synthesis of hexahydroquinoline derivatives

Entry	Catalyst	Solvent	Time (min)	Reaction cycles	Yield%	Ref.
1	Melamine trisulfonic acid (MTSA)	—	180	4	94	55
2	*n*-Fe_3_O_4_@TDI@TiO_2_	—	20	6	92	12
3	Nano-ZrO_2_–SO_3_H	—	16	5	94	10
4	Nano-Fe_3_O_4_	EtOH	12	—	91	56
5	**Fe**_**3**_ **O**_**4**_ **@SiO**_**2**_ **@RF–SO**_**3**_ **H**	**—**	**15**	**10**	**>98**	**This work**

### 3.6. Fe_3_O_4_@SiO_2_@RF–SO_3_H stability

**Fig. 6:**
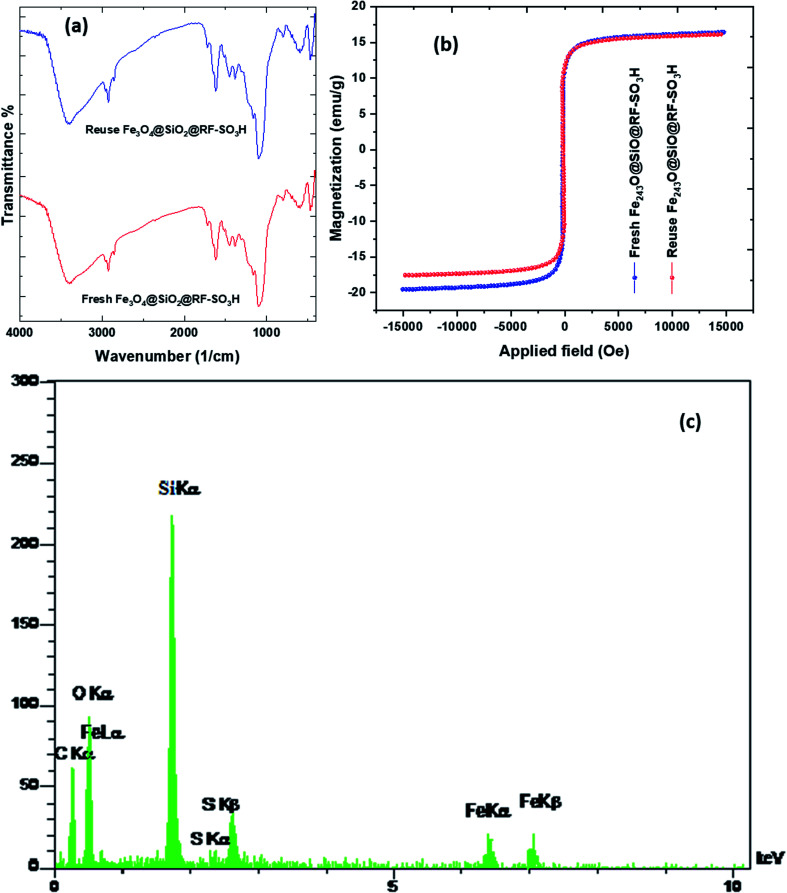
FTIR spectra (a) and VSM analysis (b) of the Fe_3_O_4_@SiO_2_@RF–SO_3_H after and before used as the catalyst and EDS spectrum of the recovered Fe_3_O_4_@SiO_2_@RF–SO_3_H after seventh reaction cycle and (c).

To study the structure and confirm the stability of the recovered catalyst, FTIR, EDS, and VSM results of the recycled Fe_3_O_4_@SiO_2_@RF–SO_3_H were investigated. The FTIR spectra results of reused catalyst indicated that the Fe_3_O_4_@SiO_2_@RF–SO_3_H structure remained intact during the hexahydroquinolines preparation reaction. The VSM analysis of recovered catalyst did not show a significant change in the saturation magnetization as both the fresh and recovered catalysts had the saturation magnetization of ∼17 emu g^−1^ (Fig. 6b). By comparing the EDS results of fresh and recovered catalysts, it can be observed that all elements in the fresh catalyst (such as Fe, Si, O, C, and S) are present in the recovered catalyst, thus indicating the stability of the Fe_3_O_4_@SiO_2_@RF–SO_3_H structure during the reaction (Fig. 6c). These results reveal the stability of the Fe_3_O_4_@SiO_2_@RF–SO_3_H structure and indicate the high activity of the Fe_3_O_4_@SiO_2_@RF–SO_3_H catalyst after recovery.

### 3.7. Fe_3_O_4_@SiO_2_@RF–SO_3_H-catalyzed hexahydroquinolines synthesis mechanism

**Scheme 1:**
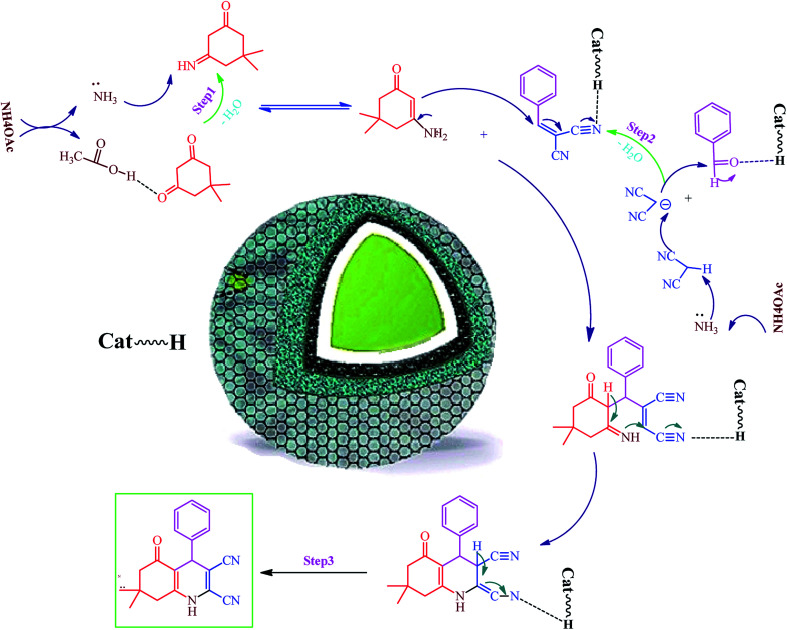
Proposed mechanism of Fe_3_O_4_@SiO_2_@RF–SO_3_H catalyzed hexahydroquinolines synthesis.

Hexahydroquinoline preparation reaction occurs in three stages. (i) The addition of ammonium acetate to the dimedone and the formation of enaminone (enaminone possesses the nucleophilic characters of enamine and enone).^57^ At this stage, ammonium acetate serves as a source of hydrogen and activates the carbonyl group from dimedone and malononitrile to form enaminone.^57^ (ii) A Knoevenagel reaction between the benzaldehyde and the malononitrile gives rise to the formation of arylidenemalononitrile (the ammonium acetate also acts as a base source and converts malononitrile into a nucleophile by removing hydrogen from the malononitrile).^58^ (iii) A Michael addition reaction involving the intramolecular cyclization between the enaminone and arylidenemalononitrile; water elimination under this condition results in the formation of the final product (Scheme 1). Owing to its acidic hydrogen, carbonyl, and cyanide groups, Fe_3_O_4_@SiO_2_@RF–SO_3_H can be activated in three different steps and enhance the activity of intermediates in each step (Scheme 1).

## 4. Conclusions

In this study, a new core-double shell nanostructure of Fe_3_O_4_@SiO_2_@RF@SO_3_H nanocatalyst was successfully synthesized and characterized using FT-IR, VSM, SEM, TEM, EDAX, TGA, and XRD analyses. FT-IR, EDAX, and TGA showed successful stabilization of sulfuric acid acidic groups on the Fe_3_O_4_@SiO_2_@RF surface. FESEM and TEM analyses confirmed the spherical core-double-shell structure of the products. This new nanocatalyst was successfully applied in a one-step reaction to synthesize the hexahydroquinoline derivatives with high purity and yield. Fe_3_O_4_@SiO_2_@RF@SO_3_H nanocatalyst was simply recycled and reused 10 times without significant reduction in its efficiency. Moreover, the efficiency of this new nanocatalyst was compared with the previously reported catalysts, which indicated shorter reaction time, higher yields, solvent-free conditions, and higher reaction cycle of the proposed nanocatalyst. Finally, EDAX and VSM results of the recycled Fe_3_O_4_@SiO_2_@RF@SO_3_H confirmed its high stability.

## Conflicts of interest

There is no conflicts of interest.

## Supplementary Material

## References

[cit1] Kumar S., Sharma P., Kapoor K. K., Hundal M. S. (2008). Tetrahedron.

[cit2] Schade D., Lanier M., Willems E., Okolotowicz K., Bushway P., Wahlquist C., Gilley C., Mercola M., Cashman J. R. (2012). J. Med. Chem..

[cit3] Wang L.-M., Sheng J., Zhang L., Han J.-W., Fan Z.-Y., Tian H., Qian C.-T. (2005). Tetrahedron.

[cit4] Heravi M. M., Bakhtiari K., Javadi N. M., Bamoharram F. F., Saeedi M., Oskooie H. A. (2007). J. Mol. Catal. A: Chem..

[cit5] Cherkupally S. R., Mekala R. (2008). Chem. Pharm. Bull..

[cit6] Maheswara M., Siddaiah V., Damu G. L. V., Rao C. V. (2006). ARKIVOC.

[cit7] Donelson J. L., Gibbs R. A., De S. K. (2006). J. Mol. Catal. A: Chem..

[cit8] Reddy C. S., Raghu M. (2008). Chin. Chem. Lett..

[cit9] Surasani R., Kalita D., Rao A. D., Yarbagi K., Chandrasekhar K. (2012). J. Fluorine Chem..

[cit10] Amoozadeh A., Rahmani S., Bitaraf M., Abadi F. B., Tabrizian E. (2016). New J. Chem..

[cit11] Safaei-Ghomi J., Aghagoli R., Shahbazi-Alavi H. (2018). Z. Naturforsch. B.

[cit12] Tabrizian E., Amoozadeh A. (2016). Catal. Sci. Technol..

[cit13] Lu A. H., Salabas E. e. L., Schüth F. (2007). Angew. Chem., Int. Ed..

[cit14] Gao J., Gu H., Xu B. (2009). Acc. Chem. Res..

[cit15] Kang Y., Zhou L., Li X., Yuan J. (2011). J. Mater. Chem..

[cit16] Jun Y.-w., Huh Y.-M., Choi J.-s., Lee J.-H., Song H.-T., Kim S., Kim S., Yoon S., Kim K.-S., Shin J.-S. (2005). J. Am. Chem. Soc..

[cit17] Jin C., Wang Y., Tang H., Wei H., Liu X., Wang J. (2014). J. Physic. Chem. C.

[cit18] Vestal C. R., Zhang Z. J. (2003). Nano Lett..

[cit19] Huang G., Zhang C., Li S., Khemtong C., Yang S.-G., Tian R., Minna J. D., Brown K. C., Gao J. (2009). J. Mater. Chem..

[cit20] Taheri S., Veisi H., Hekmati M. (2017). New J. Chem..

[cit21] Yang P., Xu Y., Chen L., Wang X., Zhang Q. (2015). Langmuir.

[cit22] Liu X., Li S., Mei J., Lau W.-M., Mi R., Li Y., Liu H., Liu L. (2014). J. Mater. Chem. A.

[cit23] Shao Y., Zhou L., Bao C., Wu Q., Wu W., Liu M. (2016). New J. Chem..

[cit24] Choma J., Jamioła D., Augustynek K., Marszewski M., Jaroniec M. (2012). Chem. Commun..

[cit25] Wu S.-H., Mou C.-Y., Lin H.-P. (2013). Chem. Soc. Rev..

[cit26] Liu J., Qiao S. Z., Liu H., Chen J., Orpe A., Zhao D., Lu G. Q. (2011). Angew. Chem..

[cit27] Zhang Q., Lee I., Joo J. B., Zaera F., Yin Y. (2012). Acc. Chem. Res..

[cit28] Zhao M., Deng K., He L., Liu Y., Li G., Zhao H., Tang Z. (2014). J. Am. Chem. Soc..

[cit29] Li Y., Jin J., Wang D., Lv J., Hou K., Liu Y., Chen C., Tang Z. (2018). Nano Res..

[cit30] Lu J., Zhou W., Wang L., Jia J., Ke Y., Yang L., Zhou K., Liu X., Tang Z., Li L. (2016). ACS Catal..

[cit31] Wang X., Fan L., Gong D., Zhu J., Zhang Q., Lu B. (2016). Adv. Funct. Mater..

[cit32] Zhang Z., Wang F., An Q., Li W., Wu P. (2015). J. Mater. Chem. A.

[cit33] Xu M., Chen D., Huang P., Wan Z., Zhou Y., Ji Z. (2016). J. Mater. Chem. C.

[cit34] Majhi S. M., Rai P., Yu Y.-T. (2015). ACS Appl. Mater. Interfaces.

[cit35] Yu X. Y., Yu L., Lou X. W. (2016). Adv. Energy Mater..

[cit36] Zou R., Yuen M. F., Yu L., Hu J., Lee C.-S., Zhang W. (2016). Sci. Rep..

[cit37] Tan P., Jiang Y., Liu X.-Q., Zhang D.-Y., Sun L.-B. (2016). ACS Sustainable Chem. Eng..

[cit38] Wu T., Liu Y., Zeng X., Cui T., Zhao Y., Li Y., Tong G. (2016). ACS Appl. Mater. Interfaces.

[cit39] Purbia R., Paria S. (2015). Nanoscale.

[cit40] Fang X., Liu S., Zang J., Xu C., Zheng M.-S., Dong Q.-F., Sun D., Zheng N. (2013). Nanoscale.

[cit41] Liu R., Priestley R. D. (2016). J. Mater. Chem. A.

[cit42] Liu Y., Wang W., Chen Q., Xu C., Cai D., Zhan H. (2019). Inorg. Chem..

[cit43] Liu R., Guo Y., Odusote G., Qu F., Priestley R. D. (2013). ACS Appl. Mater. Interfaces.

[cit44] Liu R., Yeh Y.-W., Tam V. H., Qu F., Yao N., Priestley R. D. (2014). Chem. Commun..

[cit45] Wang M., Ni Y., Liu A. (2017). ACS Omega.

[cit46] Zhang Y., Yang Y., Duan H., Lü C. (2018). ACS Appl. Mater. Interfaces.

[cit47] Amoozadeh A., Golian S., Rahmani S. (2015). RSC Adv..

[cit48] Wu Z., Chen C., Wang L., Wan H., Guan G. (2016). Ind. Eng. Chem. Res..

[cit49] Veisi H., Taheri S., Hemmati S. (2016). Green Chem..

[cit50] Yang R., Liu Y., Yan X., Liu S., Zheng H. (2016). J. Mater. Chem. A.

[cit51] Wang Y.-X., Yang J., Chou S.-L., Liu H. K., Zhang W.-x., Zhao D., Dou S. X. (2015). Nat. Commun..

[cit52] Pan J., Sun H., Yan X., Zhong W., Shen W., Zhang Y., Cheng X. (2020). Ceram. Int..

[cit53] Xie X., Chen L., Pan X., Wang S. (2015). J. Chromatogr. A.

[cit54] Zhong Y., Ni Y., Li S., Wang M. (2016). RSC Adv..

[cit55] Aswin K., Logaiya K., Sudhan P. N., Mansoor S. S. (2012). J. Taibah Univ. Sci..

[cit56] Amirheidari B., Seifi M., Abaszadeh M. (2016). Res. Chem. Intermed..

[cit57] Patil D., Chandam D., Mulik A., Jagdale S., Patil P., Deshmukh M. (2017). J. Saudi Chem. Soc..

[cit58] van Schijndel J., Molendijk D., Spakman H., Knaven E., Canalle L. A., Meuldijk J. (2019). Green Chem. Lett. Rev..

